# An efficient annotation method for image recognition of dental instruments

**DOI:** 10.1038/s41598-022-26372-y

**Published:** 2023-01-04

**Authors:** Shintaro Oka, Kazunori Nozaki, Mikako Hayashi

**Affiliations:** 1 Joint Research Department for Oral Data Science, Osaka University Dental Hospital, Suita, Japan; 2 Division for Medical Information, Osaka University Dental Hospital, Suita, Japan; 3 Department of Restorative Dentistry and Endodontology, Osaka University Graduate School of Dentistry, Suita, Japan

**Keywords:** Dental equipment, Dental education, Biomedical engineering, Computer science

## Abstract

To prevent needlestick injury and leftover instruments, and to perform efficient dental treatment, it is important to know the instruments required during dental treatment. Therefore, we will obtain a dataset for image recognition of dental treatment instruments, develop a system for detecting dental treatment instruments during treatment by image recognition, and evaluate the performance of the system to establish a method for detecting instruments during treatment. We created an image recognition dataset using 23 types of instruments commonly used in the Department of Restorative Dentistry and Endodontology at Osaka University Dental Hospital and a surgeon’s hands as detection targets. Two types of datasets were created: one annotated with only the characteristic parts of the instruments, and the other annotated with the entire parts of instruments. YOLOv4 and YOLOv7 were used as the image recognition system. The performance of the system was evaluated in terms of two metrics: detection accuracy (DA), which indicates the probability of correctly detecting the number of target instruments in an image, and the average precision (AP). When using YOLOv4, the mean DA and AP were 89.3% and 70.9%, respectively, when the characteristic parts of the instruments were annotated and 85.3% and 59.9%, respectively, when the entire parts of the instruments were annotated. When using YOLOv7, the mean DA and AP were 89.7% and 80.8%, respectively, when the characteristic parts of the instruments were annotated and 84.4% and 63.5%, respectively, when the entire parts of the instruments were annotated. The detection of dental instruments can be performed efficiently by targeting the parts characterizing them.

## Introduction

As several instruments and devices are used in medical care, they should be operated correctly and safely. However, due to staff shortages and fluctuations in the number of patients, it is extremely difficult to ensure thorough management of instruments and equipment. Therefore, incidents affecting the lives of patients and the quality of medical care are likely to occur in the general operations of healthcare workers. A specific example of a life-threatening incident is leaving instruments or gauze inside a patient’s body after a surgical operation^1^. A needlestick injury (NSI) is one of the most frequent medical incidents in dental treatment, and an NSI can occur not only during treatment but also during cleaning procedures. NSIs tend to occur intraoperatively in oral and periodontal surgery, whereas, in general dentistry, NSIs tend to occur during cleanup due to various treatment methods and frequent changes during instrument preparation^2^. Thus, to prevent NSIs, not only the surgeon or assistant, but also the cleaner, must be aware of all available instruments. In dentistry, a variety of instruments are used according to the nature of the treatment. For example, in caries treatment, an excavator is used to remove caries, and a composite instrument is used to fill composite resin; in root canal treatment, an excavator is used to remove temporary seals, and a reamer is used to form root canals; in periodontal treatment, a probe is used to measure periodontal pockets, and a scaler is used to remove calculus. It is critical that these instruments are well prepared and that their presence is known before treatment for smooth and safe treatment. In other words, to prevent medical accidents and improve the efficiency of dental treatment, it is necessary to monitor changes in the number of instruments present during treatment in detail and in real time and to implement operations that use this information.

In recent years, various technologies based on deep learning (DL)^3^ have been used in various fields. One of such technologies is object detection via image recognition^4–7^ and semantic segmentation^8,9^. Object detection is the estimation of an area in an image, where the target object exists, and semantic segmentation is the estimation of the object type for each pixel in an image. Both technologies use a computational method called convolutional neural networks (CNNs)^10^. DL-based image recognition has been used for human safety. For example, object detection is used in automobiles for collision prevention and automatic driving^11,12^ based on detection of vehicles, people, and road signs and used in trains for detection of passengers falling from platforms^13^. Object detection is also used in the medical field, for instance, to detect lesions using endoscope images^14^ and to detect abnormal areas in X-ray images^15^. Semantic segmentation is also used to extract instruments in endoscopic images^16^.

According to a study on medical instrument detection during a procedure, color codes and RFIDs have already been used to detect the presence of instruments^17,18^, but their introduction cost and maintenance burden have become an issue^19,20^. In addition, many dental instruments are small, and it is often difficult to affix color codes or attach RFIDs. Shape recognition based on edge detection using the Canny filter that do not require these methods, for instruments used in otolaryngology surgery was 84.9%^21^. Simple shape recognition via contour extraction has a limitation of being unable to detect instruments because their contours change when they overlap. Therefore, although instrument recognition via contour extraction is effective in operating rooms and other environments, where operating room nurses can arrange instruments, it is unsuitable in a general dental practice, where a surgeon inserts in and removes instruments as instruments tend to overlap. In addition, training data for image recognition systems are annotated with rectangles to detect targets; however, since instruments overlap in dental treatment, there is a high possibility that another instrument may exist within a rectangle when annotating instruments with rectangles, making efficient training difficult.

Therefore, in this study, to realize real-time monitoring of instrument selection during dental treatment, we constructed a system that detects instruments placed on a tray during dental treatment using a CNN and compared the detecting accuracy of the instruments for different annotation methods.

## Methods

In this study, to clarify the difference in the detection accuracy (DA) of instruments due to different annotation methods, we annotated images taken in a laboratory and clinic through two different approaches to create two training datasets for image recognition. The two datasets were created by annotating only parts characterizing the target instruments and by annotating the entire target instruments, respectively. The image recognition system was trained using each dataset, the weights obtained were used to detect instruments in a clinic, and the results were compared and evaluated. The DL-based object detection software YOLOv4^6^ and YOLOv7^7^ were used as the object detection method.

### Two types of annotation methods to create datasets for training and evaluation

In DL-based object detection, the labels and coordinates of the bounding boxes (BBs) of a target object in an image are estimated and used to train the detector. Therefore, to obtain images used for training and estimating the detector, a device was developed to capture images of a paper tray (size: 16 cm × 25 cm) on which instruments are placed during an actual dental procedure.

**Figure 1:**
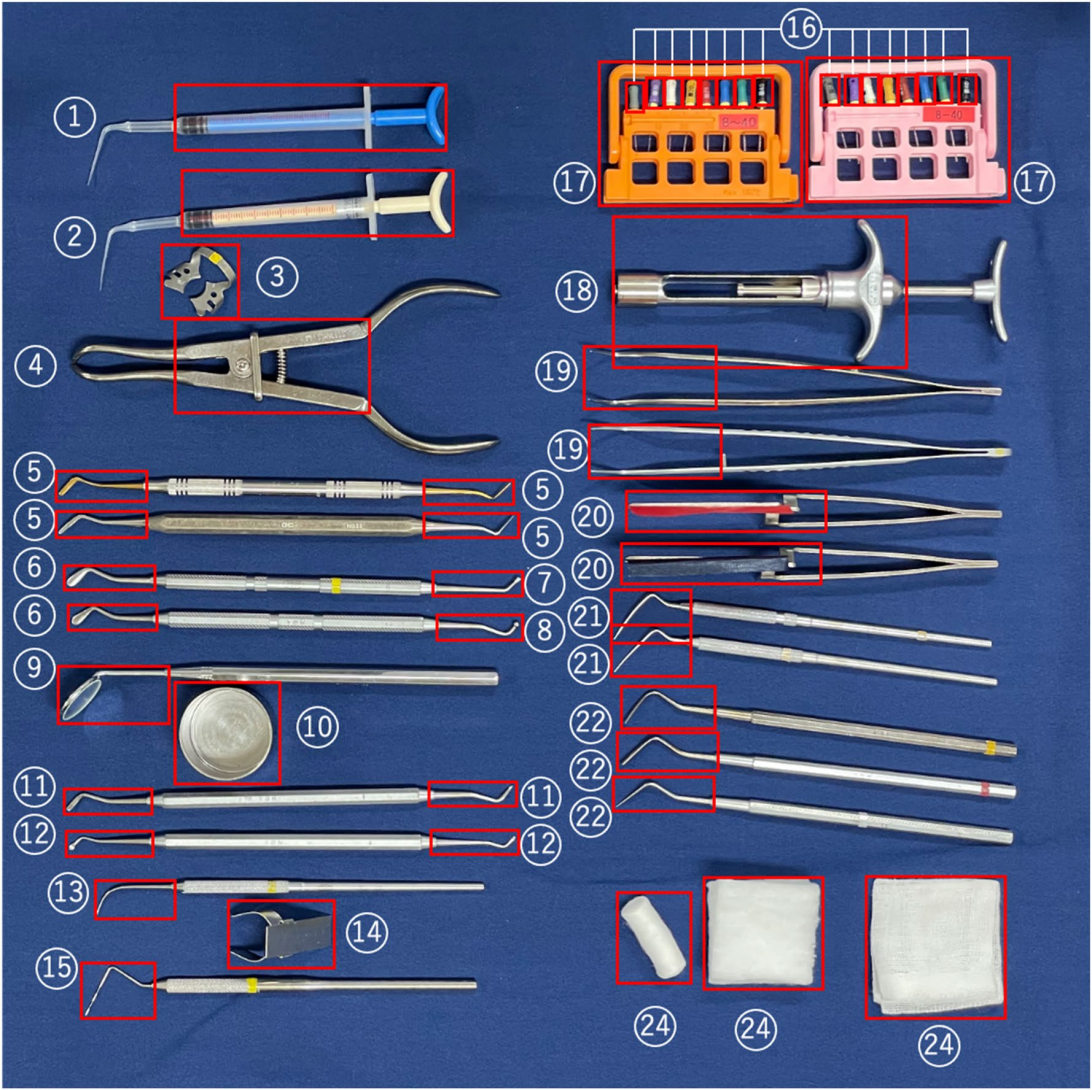
23 Types of instruments/objects targeted for image recognition for the “instrument-specific part,” in the case of a twin-headed instrument, each double-headed part was treated as a separate type of instrument. The red rectangles indicate the respective “instrument-specific part.” For instruments (6), (7), and (8), since different combinations constitute a single instrument, they were treated as the same instrument when labeling the entire part of the instrument. ①canal_syringe_blue ②canal_syringe_white ③clamp* ④clamp_forceps ⑤composite_instrument ⑥condenser ⑦condenser_disk ⑧condenser_round ⑨dental_mirror ⑩dish* ⑪excavator ⑫excavator_spoon ⑬explorer ⑭finger_ruler* ⑮probe ⑯reamer*⑰reamer_guard* ⑱syringe ⑲tweezers ⑳articulatin_paper_holder spreader plugger hand*(omitted) cotton*. An instrument with an asterisk (*) in its name denotes an instrument whose entire part has been annotated using the two annotation methods.

The device was equipped with a Raspberry Pi 3 Model B (Raspberry Pi Foundation, Cambridge, USA) and a Raspberry Pi 3 Model B (Raspberry Pi Foundation, Cambridge, USA) to capture images of the tray and its surroundings in H.264 format, 1920 pixels wide by 1080 pixels high, 25-fps frame rate, and 16.67 million color resolutions. This device can be fixed to a dental treatment table with a digital camera stand (Hakuba Photo Industry, Tokyo, Japan). In this study, images of 23 types of instruments/objects commonly used in the Department of Restorative Dentistry and Endodontology, Osaka University Dental Hospital, and a surgeon’s hands were used for image recognition (Fig. 1). From August 13, 2018, to September 25, 2018, the treatment table was photographed 64 times using this device during the treatment of consenting patients at the Hospital, and 508 images without duplication were selected by eye examination. Since the number of images that can be taken in the clinic is limited to the number of images that can be taken in a clinic room during an actual examination, we used an iPhone7 (Apple, California, USA) to capture 1–3 of the 23 different instruments on the tray, obtaining 1425 images, which were augmented to create 1943 images used in this study (Table 1).

**Table 1:** Number of images in the dataset used for training and evaluation.

	Dataset	For training	For evaluation
Clinic	Period	8/13/2018 ~ 9/25/2018	9/26/2018 ~ 1/22/2020
	Times of treatments	64	98
	Number of images	508	200
Labo	Number of images	1425	

**Figure 2:**
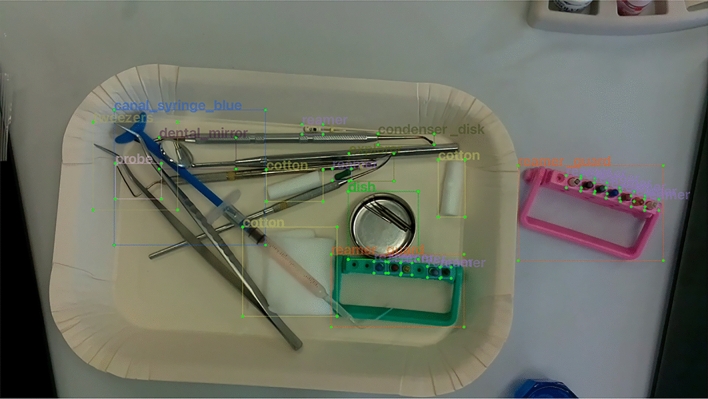
Example of labeling of instrument-specific part (AA). Only the characteristic parts of the instrument to be detected were surrounded by BBs and labeled to record the type of instrument.

**Figure 3:**
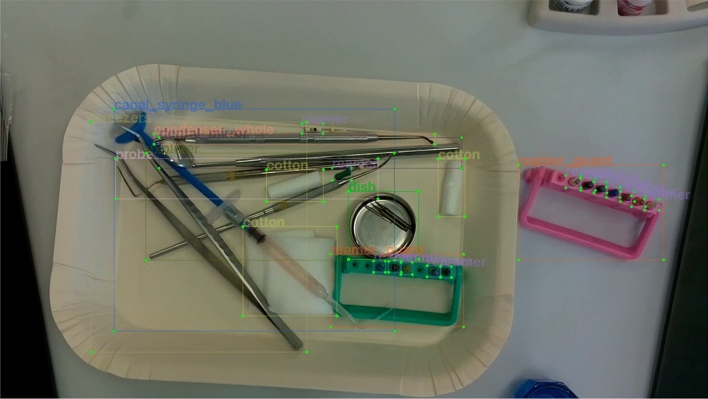
Example of labeling of entire part of instrument (AB). The entire parts of instruments to be detected were surrounded by BBs and labeled to record the type of instrument.

For the 1943 images used for training, the types of instruments present and BB coordinate information about the instruments were labeled. Here labeling was performed using two different annotation methods. The first annotation method (Annotation A: AA) annotates an instrument-specific part (Fig. 2). The “instrument-specific part” refers to a part characterizing the instrument, excluding parts common to other instruments, e.g., the gripping part, the mirror surface at the tip of a dental mirror, or the scale at the tip of a probe, (Fig. 1). The second annotation method (Annotation B: AB) annotates the entire instrument (Fig. 3). In this method, “condenser,” “condenser_disk,” and “condenser_round” are treated as the same label. Therefore, the number of labeling types was 22. In addition, it was difficult defining some specific parts, such as “clamp*,” “dish*,” “finger_ruler*,” “reamer*,” “reamer_guard*,” “hand*,” and “cotton*,” as a characteristic part of an instrument, so, in such cases, the entire apparatus was annotated using either annotation method. LabelImg^22^ was used for labeling, realizing the training dataset.

Similarly, to create the evaluation dataset, 200 images without duplication were selected by eye examination from images taken during 98 examinations of consenting patients between September 26, 2018, and January 22, 2020, and these images were annotated (Table 2).

**Table 2:** Breakdown of the number of labels for each instrument in the training and evaluation datasets.

Annotaion method	Inbstrument-specific part (AA)				Entire part of instrument (AB)			
Dataset	For training			For evaluation	For training			For evaluation
Type of instrument	Total	(Labo)	(Clinic)	Clinic	Total	(Labo)	(Clinic)	Clinic
Canal_syringe_blue	480	(130)	(350)	111	481	(130)	(351)	111
Canal_syringe_white	224	(130)	(94)	55	224	(130)	(94)	55
Clamp*	640	(640)	(10)	7	640	(640)	(10)	7
Clamp_forceps	135	(129)	(7)	8	137	(130)	(9)	9
Composite_instrument	263	(261)	(2)	44	132	(131)	(1)	26
Condenser	483	(268)	(214)	71	524	(257	(267)	80
Condenser_disk	344	(128)	(206)	52	–	–	–	–
Condenser_round	148	(127)	(21)	11	–	–	–	–
Dental_mirror	603	(130)	(473)	167	644	(130)	(514)	175
Dish*	324	(70)	(254)	86	324	(70)	(254)	86
Excavator	260	(260)	(0)	40	131	(131)	(0)	25
Excavator_spoon	265	(260)	(5)	35	135	(131)	(4)	18
Explorer	530	(130)	(400)	177	542	(130)	(412)	178
Finger_ruler*	386	(130)	(256)	75	386	(130)	(256)	75
Probe	536	(130)	(406)	179	544	(130)	(414)	179
Reamer*	3986	(840)	(3146)	1576	3986	(840)	(3146)	1576
Reamer_guard*	669	(140)	(529)	241	669	(140)	(529)	241
Syringe	189	(130)	(59)	34	190	(130)	(60)	34
Tweezers	584	(130)	(453)	189	624	(130)	(493)	199
Articulating_paper_holder	138	(130)	(9)	25	138	(130)	(9)	26
Spreader	316	(259)	(56)	10	316	(259)	(56)	10
Plugger	330	(260)	(70)	12	333	260	(73)	15
Hand*	317	(25)	(292)	27	317	(25)	(292)	27
Cotton*	1552	(0)	(1552)	641	1552	(0)	(1552)	641

### Training and evaluation of image recognition system

YOLOv4^6^ and YOLOv7^7^ were used as the image recognition system. They are one-stage detector that estimates the position and label of an existing object using a single CNN network.

For the parameters of the YOLOv4 neural network, the input size was changed to (832 × 832). For AA, the number of outputs was changed to 24, and for AB, the number of outputs was changed to 22. YOLOv4 performs object detection using a predetermined size anchor box. The appropriate size of the anchor box for training and inference differs between annotating a specific part of an instrument and annotating the entire instrument because the size of the target object differs between the two cases. Therefore, we used the k-means method to calculate the appropriate anchor box size based on the size of the BB in each image^6^. This resulted in anchor boxes of {(17, 23), (21, 39), (78, 38), (76, 71), (74, 117), (118, 187), (210, 118), (228, 260), (360, 568)} for AA, {(16, 23), (26, 26), (17, 40), (104, 53), (69, 106), (138, 196), (364, 92), (381, 220), (342, 426)} for AB.

For the parameters of the YOLOv7 neural nerwork, we used YOLOv7-E6 model and the input size was changed to (1280 × 1280), and other parameters were left at default.

To evaluate the accuracy of the detection of the number of instruments present in a clinic using the trained image recognition system, the number of each instrument detected via image recognition was set as true if it was correct and false if otherwise, and the percentage of true recognition for each instrument was calculated as the DA. In addition, as a performance evaluation metric of the image recognition system, average precision (AP) at the intersection over union (IoU) = 50% was obtained for each instrument using the same method as the PASCAL VOC Challenge^23^.

For “condenser,” “condenser_disk,” and “condenser_round” in AA, the results were averaged and summarized as “condenser.”

A desktop PC with Intel Xeon Gold 6226R CPU, 96 GB RAM, NVIDIA Quadro RTX6000 GPU, and Ubuntu 18.04 OS was used for training and evaluating YOLOv4 and YOLOv7.

This study was conducted following the Ethics Review Committee approval (H29-E23) of the Osaka University Graduate School of Dentistry and Dental Hospital, and was conducted in accordance with the “Ethical Guidelines for Medical and Biological Research Involving Human Subjects”. Although the data obtained in this study do not contain identifying information, the conditions of the instruments during the examination were photographed only after explaining the study to the patients and obtaining their informed consent in advance.

## Results

Table 3 shows the DA and DA change for each instrument for the two annotation methods (AA and AB), listed in descending order when using YOLOv4. The mean DA of instruments for AA was 89.3%, whereas that for AB was 85.3%, decreasing the mean DA by 4.0%. The six instruments for which changing the annotation method from AA to AB improved DA were “dish*,” “excavator_spoon,” “cotton*,” “tweezers,” “reamer_guard*” and “spreader,” and otherwise, DA remained the same or declined.

**Table 3:** DA of each instrument and changes according to annotation method (YOLOv4).

Inbstrument-specific part (AA)		Entire part of instrument (AB)		Change in DA from AA to AB	
Type of instrument	DA	Type of instrument	DA	Type of instrument	Change of DA
Hand*	1.000	Hand*	0.995	Dish*	0.060
Clamp*	0.990	Clamp*	0.990	Excavator_spoon	0.045
Syringe	0.975	Syringe	0.970	Cotton*	0.030
Clamp_forceps	0.970	Reamer_guard*	0.965	Tweezers	0.015
Reamer_guard*	0.960	Spreader	0.965	Reamer_guard*	0.005
Canal_syringe_white	0.960	Clamp_forceps	0.955	Spreader	0.005
Spreader	0.960	Plugger	0.935	Clamp*	0.000
Plugger	0.950	Articulating_paper_holder	0.910	Reamer*	− 0.005
Dental_mirror	0.940	Excavator_spoon	0.905	Syringe	− 0.005
Articulating_paper_holder	0.930	Canal_syringe_white	0.890	Hand*	− 0.005
Canal_syringe_blue	0.925	Canal_syringe_blue	0.885	Composite_instrument	− 0.010
Finger_ruler*	0.915	Excavator	0.875	Excavator	− 0.010
Mean	0.893	Finger_ruler*	0.870	Plugger	− 0.015
Excavator	0.885	Composite_instrument	0.870	Clamp_forceps	− 0.015
Condenser	0.882	Mean	0.853	Articulating_paper_holder	− 0.020
Composite_instrument	0.880	Dish*	0.850	Canal_syringe_blue	− 0.040
Probe	0.865	Reamer*	0.845	Mean	− 0.040
Excavator_spoon	0.860	Tweezers	0.795	Finger_ruler*	− 0.045
Reamer*	0.850	Condenser	0.795	Canal_syringe_white	− 0.070
Dish*	0.790	Dental_mirror	0.735	Condenser	− 0.087
Tweezers	0.780	Cotton*	0.725	Explorer	− 0.175
Explorer	0.715	Explorer	0.540	Dental_mirror	− 0.205
Cotton*	0.695	Probe	0.495	Probe	− 0.370

Table 4 shows the AP and AP change for each instrument for the two annotation methods (AA and AB), listed in descending order when using YOLOv4. The mean AP of instruments for AA was 70.9%, whereas that for AB was 59.9%, decreasing the mean AP by 11.0%. The six instruments with improved AP were “clamp_forceps,” “tweezers,” “dish*,” “reamer_guard*,” “clamp*” and “syringe,” and otherwise, the DA remained the same or declined.

**Table 4:** AP of each instrument and changes according to annotation method (YOLOv4).

Inbstrument-specific part (AA)		Entire part of instrument (AB)		Change in AP from AA to AB	
Type of instrument	AP	Type of instrument	AP	Type of instrument	Change of AP
Hand*	1.000	Hand*	1.000	Clamp_forceps	0.081
Dish*	0.990	Dish*	0.996	Tweezers	0.044
Reamer*	0.980	Reamer*	0.979	Dish*	0.006
Reamer_guard*	0.967	Reamer_guard*	0.971	Reamer_guard*	0.004
Dental_mirror	0.954	Cotton*	0.913	Clamp*	0.004
Cotton*	0.913	Syringe	0.906	Syringe	0.001
Canal_syringe_blue	0.905	Clamp*	0.893	Hand*	0.000
Syringe	0.905	Tweezers	0.852	Cotton*	0.000
Clamp*	0.889	Canal_syringe_blue	0.838	Reamer*	− 0.001
Probe	0.881	Dental_mirror	0.836	Finger_ruler*	− 0.022
Canal_syringe_white	0.866	Finger_ruler*	0.827	Excavator_spoon	− 0.057
Finger_ruler*	0.849	Canal_syringe_white	0.684	Canal_syringe_blue	− 0.067
Tweezers	0.808	Mean	0.599	Articulating_paper_holder	− 0.103
Explorer	0.769	Probe	0.554	Mean	− 0.110
Mean	0.709	Condenser	0.536	Dental_mirror	− 0.118
Condenser	0.706	Explorer	0.483	Composite_instrument	− 0.170
Spreader	0.651	Articulating_paper_holder	0.362	Condenser	− 0.170
Articulating_paper_holder	0.465	Clamp_forceps	0.289	Canal_syringe_white	− 0.182
Plugger	0.368	Spreader	0.141	Excavator	− 0.238
Excavator	0.298	Excavator	0.060	Explorer	− 0.286
Clamp_forceps	0.208	Plugger	0.054	Plugger	− 0.314
Composite_instrument	0.170	Composite_instrument	0.000	Probe	− 0.327
Excavator_spoon	0.057	Excavator_spoon	0.000	Spreader	− 0.510

Table 5 shows the DA and DA change for each instrument for the two annotation methods (AA and AB), listed in descending order when using YOLOv7. The mean DA of instruments for AA was 89.7%, whereas that for AB was 84.4%, decreasing the mean DA by 5.3%. The three instruments for which changing the annotation method from AA to AB improved DA were “reamer_guard*,” “composite_instrument” and “excavator_spoon,” and otherwise, DA remained the same or declined.

**Table 5:** DA of each instrument and changes according to annotation method (YOLOv7).

Inbstrument-specific part (AA)		Entire part of instrument (AB)		Change in DA from AA to AB	
Type of instrument	DA	Type of instrument	DA	Type of instrument	Change of DA
Clamp_forceps	0.995	Hand*	0.990	Reamer_guard*	0.025
Syringe	0.995	Clamp_forceps	0.985	Composite_instrument	0.020
Hand*	0.990	Syringe	0.985	Excavator_spoon	0.015
Clamp	0.985	Reamer_guard*	0.975	Hand*	0.000
Canal_syringe_white	0.965	Clamp	0.955	Articulating_paper_holder	− 0.010
Spreader	0.965	Spreader	0.955	Clamp_forceps	− 0.010
Reamer_guard*	0.950	Articulating_paper_holder	0.935	Syringe	− 0.010
Articulating_paper_holder	0.945	Plugger	0.910	Spreader	− 0.010
Dish*	0.935	Excavator_spoon	0.895	Plugger	− 0.010
Canal_syringe_blue	0.925	Finger_ruler*	0.885	Finger_ruler*	− 0.025
Plugger	0.920	Composite_instrument	0.885	Excavator	− 0.025
Finger_ruler*	0.910	Canal_syringe_white	0.880	Clamp	− 0.030
Excavator	0.905	Excavator	0.880	Cotton*	− 0.040
Mean	0.897	Canal_syringe_blue	0.860	Reamer*	− 0.045
Excavator_spoon	0.880	Mean	0.844	Mean	− 0.053
Reamer*	0.875	Reamer*	0.830	Tweezers	− 0.053
Composite_instrument	0.865	Dish*	0.805	Canal_syringe_blue	− 0.065
Dental_mirror	0.865	Tweezers	0.740	Canal_syringe_white	− 0.085
Condenser	0.865	Cotton*	0.735	Dish*	− 0.130
Probe	0.845	Condenser	0.725	Condenser	− 0.140
Tweezers	0.780	Dental_mirror	0.645	Explorer	− 0.145
Cotton*	0.775	Probe	0.590	Dental_mirror	− 0.220
Explorer	0.665	Explorer	0.520	Probe	− 0.255

Table 6 shows the AP and AP change for each instrument for the two annotation methods (AA and AB), listed in descending order when using YOLOv7. The mean AP of instruments for AA was 80.8%, whereas that for AB was 63.5%, decreasing the mean AP by 17.3%. The two instruments with improved AP were “hand*” and “clamp,” and otherwise, the DA declined.

**Table 6:** AP of Each instrument and changes according to annotation method (YOLOv7).

Inbstrument-specific part (AA)		Entire part of instrument (AB)		Change in AP from AA to AB	
Type of instrument	AP	Type of instrument	AP	Type of instrument	Change of AP
Hand*	0.996	Hand*	1.000	Hand*	0.004
Clamp_forceps	0.995	Reamer*	0.980	Clamp	0.001
Reamer*	0.986	Reamer_guard*	0.980	Reamer_guard*	− 0.004
Dish*	0.985	Dish*	0.979	Reamer*	− 0.006
Syringe	0.985	Syringe	0.971	Dish*	− 0.006
Reamer_guard*	0.984	Clamp	0.945	Tweezers	− 0.006
Canal_syringe_white	0.983	Cotton*	0.924	Syringe	− 0.014
Canal_syringe_blue	0.978	Canal_syringe_blue	0.888	Cotton*	− 0.028
Dental_mirror	0.977	Dental_mirror	0.876	Canal_syringe_blue	− 0.090
Cotton*	0.952	Tweezers	0.875	Dental_mirror	− 0.101
Clamp	0.944	Finger_ruler*	0.797	Articulating_paper_holder	− 0.109
Finger_ruler*	0.927	Canal_syringe_white	0.701	Finger_ruler*	− 0.130
Probe	0.914	Clamp_forceps	0.672	Mean	− 0.173
Tweezers	0.881	Mean	0.635	Excavator_spoon	− 0.251
Mean	0.808	Probe	0.573	Canal_syringe_white	− 0.282
Explorer	0.804	Articulating_paper_holder	0.527	Explorer	− 0.310
Condenser	0.681	Explorer	0.494	Condenser	− 0.318
Excavator	0.640	Condenser	0.363	Clamp_forceps	− 0.323
Articulating_paper_holder	0.636	Spreader	0.196	Composite_instrument	− 0.338
Spreader	0.556	Composite_instrument	0.120	Probe	− 0.341
Plugger	0.498	Excavator	0.082	Spreader	− 0.360
Composite_instrument	0.458	Excavator_spoon	0.015	Plugger	− 0.487
Excavator_spoon	0.266	Plugger	0.011	Excavator	− 0.558

## Discussion

### Effectiveness of the proposed method

The accuracy of detecting instruments during otolaryngology surgery via shape recognition based on contour extraction is 84.9%^21^. The CNN used in this study extracts feature maps using a kernel for local regions to be explored from images and evaluates the similarity of feature vectors^24^. Therefore, it is more suitable for detecting deformable objects such as “clamp_forceps” and “syringes” than shape recognition because CNN-based image recognition is robust to deformation and size changes. Although the evaluation method used in this study is different from that used in previous studies and thus cannot be uniformly evaluated, the number of instruments present was detected with a certain degree of accuracy, even when the instrumentation nurse did not align those instruments and when they overlapped. This indicates that the proposed CNN-based instrument detection method is effective in detecting dental treatment instruments placed on top of each other on a narrow tray during dental treatment.

In addition, to improve the DA of dental instruments by image recognition using a dataset containing a few images, it was shown that DA and AP were improved by explicitly annotating images of the target part of an instrument, instead of annotating images of the entire instrument for both YOLOv4 and YOLOv7. This annotation method is considered reasonable and efficient because dentists also discriminate instruments by identifying the tips where the instruments perform their function. However, since it is possible to narrow down the candidates of instrument types from the grasping part that is common to multiple instruments, it cannot be assured that excluding the common parts of each instrument from the annotation will necessarily improve DA when learning instrument types from a significant number of images.

### Relationship between DA and AP

Although AP is used as a general image recognition performance indicator, it has the problem that it cannot evaluate omissions of detection (false negatives) because its evaluation is based on the reliability and correctness of the results obtained via image recognition. Since the focus of this study was to correctly detect the number of instruments present during dental treatment, we defined a DA index to evaluate the correctness of the number of instruments detected.

This evaluation index tends to be suitable for instruments with a reasonably high frequency of occurrence: (e.g., “reamer_guard,” “tweezers,” “probe,” “explorer,” and “dental_mirror”). However, there is a problem that the number of true negatives for infrequently occurring instruments is high, resulting in high apparent values, even if there are many false negatives. For example, instruments that appeared less than 20 times in the evaluation dataset (“clamp*,” “clamp_forceps,” “spreader,” and “plugger”) all had above-average DA of more than 90%. However, the AP of all other instruments except “clamp” in YOLOv4, “clamp” and “clamp_forceps” in YOLOv7 were below average for each annotation method. Conversely, for instruments placed in large quantities at a time, even one false positive or negative results in false detection, reducing the DA. For example, instruments with a frequency of 600 or more occurrences (“reamer” and “cotton”) in the evaluation dataset both had AP above 90%, but their DA was below average.

### Factors affecting DA and AP

Instruments included in the basic set of dental practice in the Department of Restorative Dentistry and Endodontology are “dental_mirror,” “explorer,” “tweezer,” and “probe.” The DA for most of these instruments was below the average of the results for each annotation method. However, for AP, “dental_mirror” showed a high value of 95.4% in YOLOv4 and 97.7% in YOLOv7 when annotating instrument-specific parts. This result may be due to the larger mirror surface area of “dental_mirror” and its relative area in the BB. The one-stage detector has a tradeoff with its high detection speed, which reduces the recognition accuracy of small objects^25^. Among the instruments in the basic dental set, the recognition accuracy of “explorers,” “tweezers,” and “probes” was lower. This may be because the parts characterizing the instruments were sharper and smaller, and the parts discriminating the instruments were relatively smaller when the entire body of the instrument was annotated.

On the other hand, a common trend among all types of instruments with high AP was that they had a monotonous shape and a large size in the BB. “Hand,” “dish,” “reamer,” “reamer_guard,” and “cotton” are typical examples, as there are no sharp edges as the shape of the instruments and the area of the instruments occupying the BB is large. However, when the entire part of an instrument is annotated, the shape of the BB becomes more complex, including the gripping part, and AP is considered to have decreased.

A common tendency of instruments with low AP was the small number of annotations required and shape complexity. For example, “excavator_spoon,” “composite_instrument,” “clamp_forceps” and “excavator” all had less than 300 annotations in the training data, and their shapes were also complex. Since augmenting the training dataset generally improves the training effect on image recognition systems, it is important to increase the number of training images for instruments with complex shapes.

### Effect of annotation method on DA and AP

For instruments where their entire parts were targeted for detection using both annotation methods (instruments with * appended to the instrument name), there were differences in results for both DA and AP. This may be due not only to differences in the initial anchor box size in YOLOv4 but also to differences in the number of output layers and the annotations of other instruments used as negative examples during training.

Instruments for which annotating their entire parts improved DA were “excavator_spoon,” “tweezers” and “spreader” in YOLOv4, and “composite_instrument” and “excavator_spoon” in YOLOv7. Howeger, the change was smaller than that of “dish*” in YOLOv4, and “reamer_guard*” in YOLOv7 which was not changed annotation method. Therefore, changing the method from annotating the entire part of instrument to annotating instrument-specific part improved DA. Conversely, annotating an entire instrument (“condenser,” “explorer,” “dental_ mirror,” “probe,” etc.) resulted in a larger than average decrease in DA, probably because annotating the entire body of an instrument made it difficult to detect parts that identify the instrument, as described above.

The AP of “clamp_forceps” and “tweezers” was more improved than that of “dish*” in YOLOv4. For these instruments, annotating their entire parts, rather than characteristic parts, may improve DA and allow for more efficient learning. On the other hand, for “probe,” “dental_mirror,” and “explorer,” DA decreased by more than 10%. This is a larger change than 4.5% for “finger_ruler*” in YOLOv4, 13.0% for “dish*” in YOLOv7 which did not change its annotation method. AP of all instruments was improved, except for instruments for which the annotation method was not changed, namely, “clamp_forceps,” “tweezers” and “syringe” in YOLOv4, and “clamp” in YOLOv7. Therefore, annotating an entire instrument decreases DA, which may reduce the learning efficiency.

### Medical applications and future prospects for system development

In the medical field, instruments and gauze are frequently left behind in patients’ bodies during laparotomies. When an instrument or gauze is left behind, not only reoperation is required, but in the worst-case scenario, the patient may die. To avoid such risks, it is considered reasonable that all patients should be radiographed after surgery^1^. However, a problem exists in that patients are exposed to radiation, which is essentially unnecessary. By introducing our image recognition-based method for identifying the number of instruments present in the surgical field, the risk of leaving instruments behind in a patient’s body can be mitigated and minimally invasive medical care can be provided to patients during surgery.

In addition, by analyzing the history of instrument use and procedures automatically generated from object detection results, operational efficiency can be improved, for instance, by suggesting combinations of instruments when preparing instruments necessary for a procedure, or by suggesting when to replace instruments based on their frequency of use, thereby preventing accidents involving instrument damage. This will reduce the workload of medical personnel, which in turn will improve medical safety.

## Conclusions

In this study, we developed a real-time dental instrument detection system using CNNs. When creating the dataset used to train the CNN, it was found that by annotating only those parts of the dataset that characterize medical instruments rather than annotating the entire body of the instruments, the mean DA, the percentage of the number of instruments correctly detected, was improved by 4.0–5.3%, and the mean AP was improved by 11.0–17.3%.

## Data Availability

The datasets used during the current study available from the corresponding author on reasonable request.
